# Construction and validation of a prognostic model for colon adenocarcinoma based on bile acid metabolism-related genes

**DOI:** 10.1038/s41598-023-40020-z

**Published:** 2023-08-05

**Authors:** Qinghua Luo, Ping Zhou, Shuangqing Chang, Zhifang Huang, Xuebo Zeng

**Affiliations:** 1 Department of Anorectal Surgery, Wuyi Hospital of Traditional Chinese Medicine, Jiangmen, China; 2 Department of Anorectal Surgery, Jiangxi Hospital of Integrated Traditional Chinese and Western Medicine, Nanchang, China; 3 Department of Brain Diseases, Shenzhen Pingle Orthopaedic Hospital, Shenzhen, China

**Keywords:** Cancer microenvironment, Cancer, Cancer models

## Abstract

Colon adenocarcinoma (COAD), one of the common clinical cancers, exhibits high morbidity and mortality, and its pathogenesis and treatment are still underdeveloped. Numerous studies have demonstrated the involvement of bile acids in tumour development, while the potential role of their metabolism in the tumor microenvironment (TME) has not been explored. A collection of 481 genes related to bile acid metabolism were obtained, and The Cancer Genome Atlas-based COAD risk model was developed using the least absolute shrinkage selection operator (LASSO) regression analysis. The Gene Expression Omnibus dataset was used to validate the results. The predictive performance of the model was verified using column line plots, principal component analysis and receiver operating characteristic curves. Then, we analysed the differences between the high- and low-risk groups from training set based on clinical characteristics, immune cell infiltration, immune-related functions, chemotherapeutic drug sensitivity and immunotherapy efficacy. Additionally, we constructed a protein–protein interaction network to screen for target genes, which were further investigated in terms of differential immune cell distribution. A total of 234 bile acids-related differentially expressed genes were obtained between normal and tumour colon tissues. Among them, 111 genes were upregulated and 123 genes were down-regulated in the tumour samples. Relying on the LASSO logistic regression algorithm, we constructed a model of bile acid risk score, comprising 12 genes: *CPT2, SLCO1A2, CD36, ACOX1, CDKN2A, HADH, GABRD, LEP, TIMP1, MAT1A, SLC6A15* and *PPARGC1A.* This model was validated in the GEO-COAD set. Age and risk score were observed to be independent prognostic factors in patients with COAD. Genes related to bile acid metabolism in COAD were closely related to bile secretion, intestinal transport, steroid and fatty acid metabolism. Furthermore, the high-risk group was more sensitive to Oxaliplatin than the low-risk group. Finally, the three target genes screened were closely associated with immune cells. We identified a set of 12 genes (*CPT2, SLCO1A2, CD36, ACOX1, CDKN2A, HADH, GABRD, LEP, TIMP1, MAT1A, SLC6A15,* and *PPARGC1A*) associated with bile acid metabolism and developed a bile acid risk score model using LASSO regression analysis. The model demonstrated good predictive performance and was validated using an independent dataset. Our findings revealed that the bile acid risk score were independent prognostic factors in COAD patients.

## Introduction

As one of the most common malignant tumours of the human digestive system, intestinal cancer poses a significant burden to human health and the economy. Moreover, its morbidity and mortality rates have been recently increasing^1^. There are many risk factors for intestinal cancer, among which poor dietary habits, ageing, obesity, frequent smoking and lack of physical activity are the most relevant^2^. Most cases of colon adenocarcinoma (COAD) follow a pattern of adenomatous polyps, precancerous polyps and, finally, adenocarcinoma^3^. Adenomatous polyps, precancerous polyps and intramucosal carcinomas can be treated with endoscopic polypectomy, endoscopic mucosal resection and endoscopic submucosal dissection^4^. However, for non-metastatic stage II and III COAD, laparoscopic surgery is the preferred method^4^. Moreover, current clinical guidelines recommend using adjuvant chemotherapy, DNA testing and targeted therapy for advanced cases^5^. Despite the varied treatment options, individual genetic heterogeneity among patients with COAD poses a challenge in clinical practice.

Bile acids play an essential role in the function of the human body, affecting intestinal nutrient absorption and bile secretion. They also act as signalling molecules and metabolic regulators, constantly stimulating nuclear receptors and G protein-coupled receptors (GPCR) and maintaining hepatic lipid, glucose and energy stability in the body^6^. By activating bile acid regulators, steatohepatitis and fibrosis can be reversed and thus used to treat non-alcoholic steatohepatitis^7^. Moreover, abnormalities in bile acid metabolism can cause disturbances in insulin homeostasis, leading to diabetes^8^. Additionally, bile acids, the end product of cholesterol catabolism in the host liver, can potentially influence the development of inflammatory bowel disease owing to the impairment of its signalling pathways and the abnormal expression of its activating receptors FXR, GPBAR1, PXR, VDR and RORγt^9^. Bile acids are an essential component of the intestinal tract, wherein it bridges the gap between the intestinal microbiota and intestinal metabolism, regulates gastrointestinal motility, alters intestinal permeability and influences the carcinogenesis process^10^. Notably, the selective activation of FXR receptors of bile acids can effectively limit the growth of abnormal Lgr5 cells and inhibit the progression of COAD^11^. Bile acids have also been identified as carcinogenic factors, and their interaction with intestinal microorganisms can accelerate the transformation of benign adenomas into malignant tumours^12^. A high intake of high-fat diet causes elevated bile acids levels in the colon and converts colonic epithelial cells into cancer stem cells through the beta-linked protein signalling pathway^13^. A deeper understanding of the molecular mechanisms underlying COAD, especially metabolic pathways, can aid in developing novel ideas and approaches for COAD treatment^14^.

Thus, this study aims to elucidate the relationship between bile acid metabolism-related genes and COAD. We also aim to construct a predictive risk score model of bile acid metabolism using genomic data of COAD samples obtained from the cancer database. This study can provide beneficial clinical guidance, circumventing the problem of genetic heterogeneity of the patients, and aid in the development of prognostic markers, which are genes related to bile acid metabolism, for patients with COAD.

## Materials and methods

### Clinical data collection and collation

From The Cancer Genome Atlas (TCGA) database (https://portal.gdc.cancer.gov/), we downloaded raw RNA sequencing (RNA-seq) datasets and clinical information data profiles, which contained 473 COAD samples and 41 normal colorectal tissue samples. Additionally, the GSE39582 (GEO-COAD-dataset1) and GSE17538 datasets (GEO-COAD-dataset2) were obtained from the Gene Expression Omnibus (GEO) database (https://www.ncbi.nlm.nih.gov/geo/), along with their transcriptional analysis data and clinical data. Using human gene annotation files, we converted the gene IDs of the samples to the corresponding gene symbols.

### Acquisition of genes related to bile acid metabolism

In the GeneCards database (https://www.genecards.org/), we used the keyword “bile acid” to obtain the associated genes. From the obtained genes, we selected a total of 481 genes with a correlation score > 12 (Supplementary Table S1) for further analyses.

### Identification of differentially expressed genes and enrichment analysis

We screened for differential expressed genes related to bile acid metabolism using the “Deseq2” R package with the following settings: LogFC < 0.585 and FDR < 0.05. To further understand the biological characteristics and functional cellular pathways, we constructed the GO and KEGG pathways enriched with differential genes using the “clusterprofiler” R package (Version: 4.8.1) with the *p* value and corrected *p* value set at < 0.05 for statistical significance. “ggplot 2” (Version: 3.4.2) and “goplot” R packages (Version: 1.0.2) were used to visualize the enrichment analysis results.

### Construction and validation of a bile acid metabolic risk score model

Survival data were combined with the differentially expressed genes related to bile acid metabolism to screen for prognosis-related genes. The *p* values were set to < 0.05, and univariate Cox regression analysis was performed on the training set. Furthermore, we developed a predictive risk score model for predicting overall survival (OS) in COAD samples using the Least Absolute Shrinkage Selection Operator (LASSO) Cox regression analysis method. The bile acids risk score of all samples are calculated according to the equation: risk score = ∑Coef *ExpGene, where “Coef” corresponds to the non-zero regression coefficient obtained by LASSO Cox regression analysis, and “ExpGene” corresponds to the expression value of the gene in the prognostic risk score model. The median bile acid risk score of the TCGA-COAD cohort was used as the cut-off value to divide the patients with COAD into high-risk and low-risk groups. Kaplan–Meier curves were used to analyse the differences in survival prognosis between these two groups.

### Identification of receiver operating characteristic curve (ROC) and independent prognostic indicators

Using the “timeROC” package (Version: 0.4), transient ROC curves were plotted. The prognosis-related bile acid metabolism genes were identified as independent predictors of OS using the Cox proportional risk regression model. Furthermore, the relationship between risk score and clinical information was explored using the “limma” package (Version: 3.56.2), which included gender, age, pathological stage and TNM stage. *p* < 0.05 was considered statistically significant, and independent prognostic indicators were screened using univariate and multivariate Cox regression analyses.

### Construction and evaluation of the nomograms of patients with COAD

The “nomogram” package in R was used to construct a predictive model based on significant clinical parameters, which can be easily used to study the OS of each patient with COAD. To assess the predictive performance of this model, ROC curves and calibration plots were used.

### Characteristics of patients in the high- and low-risk groups

To study the tumour mutational load (TMB) of patients with COAD, we downloaded the mutation data from the TCGA database. Using the “ggpubr” package in R, correlations between patient risk scores and tumour mutation frequency in target genes were obtained. Then, immune cell infiltration files were obtained from Timer2.0 (http://timer.cistrome.org/) and the “limma” (Version: 3.56.2) and “pheatmap” R packages (Version: 1.0.12) were used to clarify and visualise the relationship between immune cell infiltration and risk score of patients from training set, respectively. The “GSVA” (Version: 1.48.2) and “GSEABase” (Version: 1.62.0) packages in R were further used to analyse the differences in immune-related functions between the high- and low-risk groups. Using the “PRRophetic” R package (Version: 0.5.1), the semi-inhibitory concentrations of drugs in the high- and low-risk groups were predicted and the drugs with differential efficacy were obtained. Additionally, the TIDE online database (http://tide.dfci.harvard.edu/) was used to predict the effect of immunotherapy in the high- and low-risk groups, with statistical significance set at *p* value < 0.05.

### Protein–protein interaction (PPI) network and target gene characteristics

Using the STRING online database (https://cn.string-db.org/), the generated interaction scores were set at > 0.40 (medium confidence level), and the differential genes between the high- and low-risk score groups were processed to map the PPI interactions. Cytoscape software was used to identify the upregulated and down-regulated genes. The CubHubba plugin (Version 3.9.1) of the Cytoscape software was used to screen the top 10 network core genes. Using the GEPIA (http://gepia.cancer-pku.cn) online database, we set the threshold at log2FC > 1 and *p* value < 0.05 to screen the highly expressed tumour-related target genes among the top 10 core genes. Furthermore, the infiltration of 22 tumour-infiltrating lymphocytes in the microenvironment of patients with COAD was analysed using the CIBERSORTx (https://cibersortx.stanford.edu/index.php) online database and the “reshape2” (Version: 1.4.4) and “ggpubr “R” packages (Version: 0.6.0). Finally, the GEPIA (http://gepia.cancer-pku.cn) online database and Spearman’s test were used to identify the correlation between target genes.

## Results

### Enrichment analysis of differential genes between tumor or normal samples

**Figure 1:**
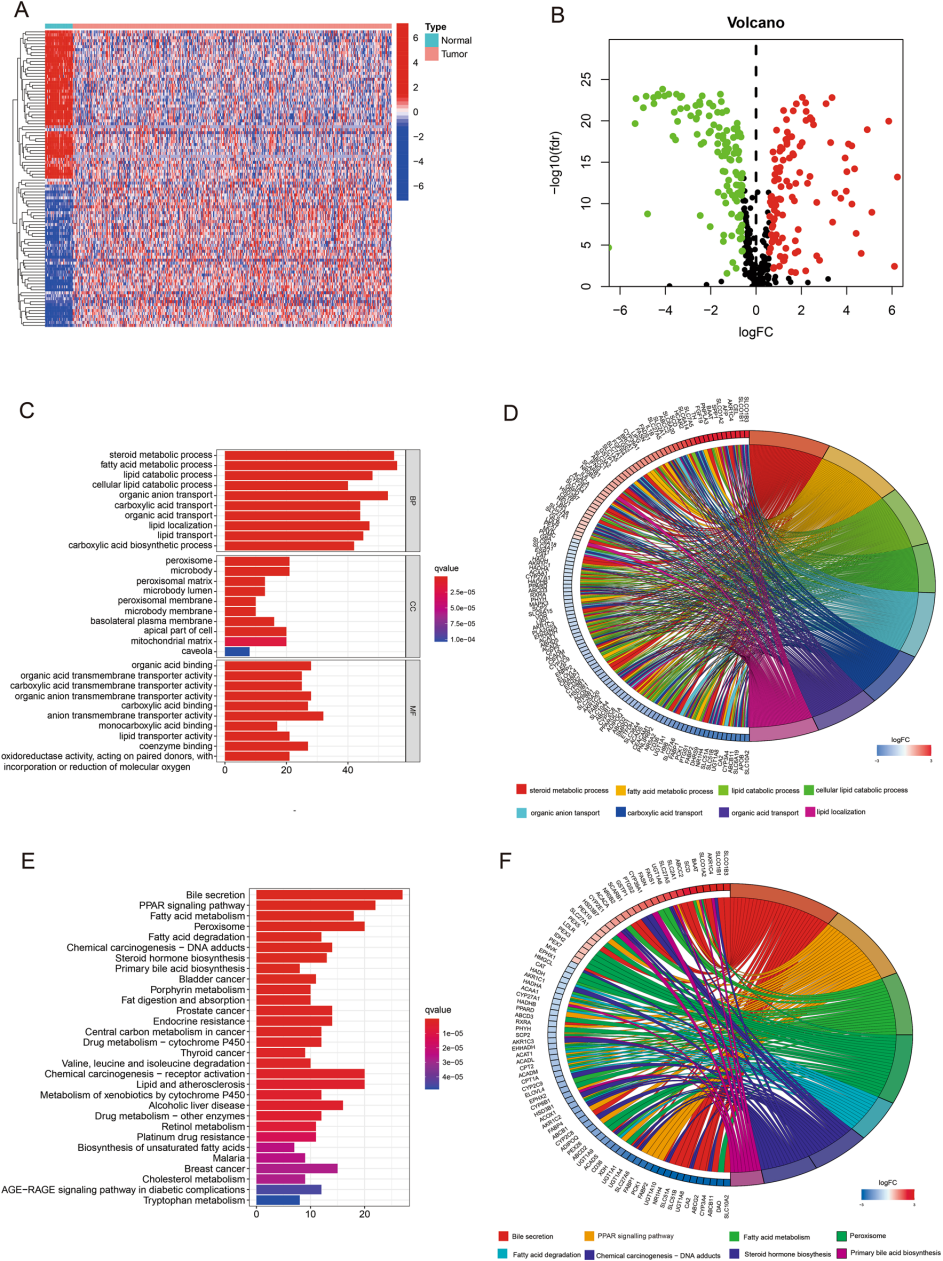
(**A**) Heat map of 234 genes in the TCGA-COAD cohort. (**B**) Volcano map of the 234 bile acid metabolism-related differential genes in the TCGA-COAD cohort. (**C**, **D**) GO analysis of bile acid metabolism-related differential genes in the TCGA-COAD cohort. (**E**, **F**) KEGG analysis of bile acid metabolism-related differential genes in the TCGA-COAD cohort.

The COAD samples and standard samples obtained from the TCGA database were analysed to obtain the genes with differential expression in bile acid metabolism-related genes with the *p* value and FDR value set to “ < 0.05” and “ < 0.585”, respectively. A total of 234 differentially expressed genes were obtained. Among them, 111 genes were upregulated and 123 genes were down-regulated in the tumour samples (Fig. 1A, B). Furthermore, GO enrichment analysis reveals that in terms of biological processes, the differential genes related to bile acid metabolism are highly enriched in fatty acid metabolic processes, steroid metabolic processes and organic anion transport. Additionally, in terms of molecular functions, they are highly enriched in the 125 most significantly up-regulated genes. The corresponding enrichment pathways are shown in Fig. 1C, D. The top 83 highly enriched genes and their interconnected enrichment pathways are presented in Fig. 1E, F. Supplementary Fig. 1A displays the KEGG enrichment pathways associated with up-regulated DEGs, while Supplementary Fig. 1B presents the KEGG enrichment pathways associated with down-regulated DEGs. Additionally, Supplementary Fig. 1C exhibits the GO enrichment pathways linked to up-regulated DEGs, while Supplementary Fig. 1D showcases the GO enrichment pathways associated with down-regulated DEGs. Thus, these findings indicate that genes related to bile acid metabolism in COAD are closely related to bile secretion, intestinal transport, steroid metabolism and fatty acid metabolism and can act as signals and receptors to influence cancer processes.

### Construction of a risk score model in the training set

**Figure 2:**
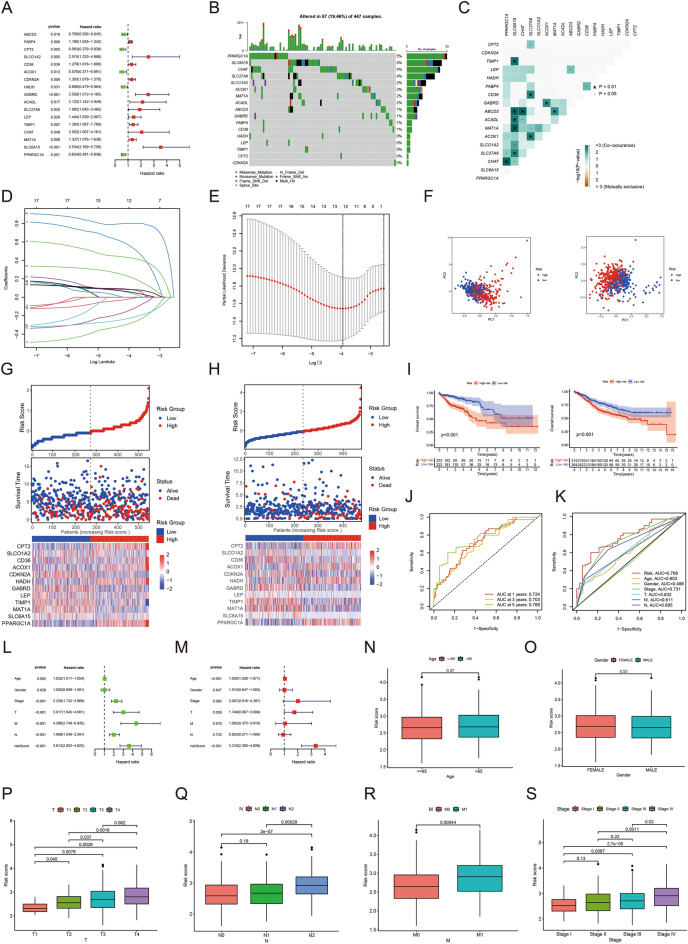
(**A**) Univariate analysis of genes related to bile acid metabolism. When the hazard ratio of a gene is > 1, it indicates that the gene is a risk factor for the corresponding tumor, and vice versa. (**B**) Gene mutations in patients with TCGA-COAD. (**C**) Correlation of mutations in 17 bile acid metabolism genes. Brown color indicates negative correlation, and blue color indicates positive correlation. *p* < 0.05, *p < 0.01. (**D**) LASSO coefficient spectrum of 17 bile acid metabolism genes. (**E**) Cross-validation of adjustment parameter selection in a proportional hazards model. (**F**) PCA based on bile acid metabolism risk scores in the TCGA-COAD cohort and GEO-COAD-dataset1. The left group represents TCGA-COAD cohort, and the right group represents GEO-COAD-dataset1. (**G**) Heatmap for the expression of six crucial genes in TCGA-COAD cohort and GEO-COAD-dataset1. The left group represents TCGA-COAD cohort, and the right group represents GEO-COAD-dataset1. (**H**) The distribution of bile acid metabolism risk scores and survival status of COAD patients with increasing bile acid metabolism risk scores. The left group represents TCGA-COAD cohort, and the right group represents GEO-COAD-dataset1. (**I**) OS by bile acid risk score in the TCGA-COAD cohort and GEO-COAD-dataset1. The left group represents TCGA-COAD cohort, and the right group represents GEO-COAD-dataset1. (**J**) AUC values at 1, 3, and 5 years in the TCGA-COAD cohort. (**K**) ROC curves of risk scores and clinical characteristics in the TCGA-COAD cohort. (**L**) Results of univariate Cox analysis in the TCGA-COAD cohort. (**M**) Multivariate Cox analysis results in the TCGA-COAD cohort. (**N** − **S**) The relationship of risk score and clinicopathological features, including age (**N**), gender (**O**), tumor invasion (**P**), lymphoid metastasis (**Q**), distal metastasis (**R**), and TNM stage (**S**).

Univariate Cox regression analysis was performed on tumour and normal samples from the TCGA-COAD cohort, wherein 17 bile acid metabolism-related genes were selected from the 234 differential bile acid metabolism genes associated with patient prognosis (Fig. 2A). Based on these 17 prognosis-related genes, we plotted the corresponding oncoplot, which revealed that 87 of the 447 samples produced mutations (19.46%) (Fig. 2B). Moreover, *PARGC1A, SLC6A15, CHAT,* and *SLC27A6* exhibited the most apparent mutations and their corresponding mutation frequencies were 5%, 4%, 4% and 4%, respectively. Additionally, the mutation frequencies of *SLCO1A2, ACOX1, MAT1A, ACADL, ABCD3, GABRD, FABP4* and *CD36* ranged from 1 to 2%. According to the visualization of co-mutations, two genes, *SLC6A15* and *ABCD3*, had more positive co-mutations than the other genes (Fig. 2C). Relying on the LASSO logistic regression algorithm, we constructed a model of bile acid risk score, comprising 12 genes: *CPT2, SLCO1A2, CD36, ACOX1, CDKN2A, HADH, GABRD, LEP, TIMP1, MAT1A, SLC6A15 and PPARGC1A* (Fig. 2D, E)*.* We calculated the risk score for each patient using the following formula: risk score = ∑(Coef *ExpGene), where EXPGENE represents the gene expression level, and Coef is derived from non-zero regression coefficients obtained through LASSO Cox regression analysis. The corresponding values of Coef for each gene are presented in Supplementary Table S2. The scores, survival status of each patient with COAD from TCGA are shown in Fig. 2G. In order to validate the prognostic model in external validation GEO-COAD-dataset1 (GSE39582), we calculate the risk score of each patient in the external validation set according to the same risk score formula we constructed. The patients in the external validation set were divided into the high-risk group and low-risk group based on the median risk score value of the training set (Fig. 2H).

In the survival analysis, the clinical prognosis was better in the low-risk group than in the high-risk group (*p* value < 0.05) (Fig. 2I). Subsequently, we further validated the model in another independent dataset, GEO-COAD-dataset2 (GSE17538). Remarkably, Kaplan–Meier analysis revealed that even in this dataset, there remained a significant survival difference between the high-risk and low-risk patient groups, further supporting the robustness and generalizability of our prognostic model. As shown in Fig. 2J, the area under the curve (AUC) values for the ROC analysis were 0.724, 0.703 and 0.768 for the 1-, 3- and 5-year OS in the TCGA cohort, respectively. Our risk model demonstrated a highly significant area under the receiver operating characteristic (ROC) curve, indicating its robustness and reliability (Fig. 2K). Univariate prognostic COX analysis of the TCGA-COAD cohort showed that age, grade, T stage, lymph node status, distal metastasis and risk score correlate with OS. However, multivariate COX analysis indicated that age and risk score were independent predictors of OS (*p* value < 0.05) (Fig. 2L, M). Comparison of the analysis scores and sample factors revealed that gender and age were not significantly different from risk scores (Fig. 2N, O), and TMN and staging showed a positive correlation with risk scores (*p* value < 0.05) (Fig. 2P–S).

### Construction and evaluation of nomograph

**Figure 3:**
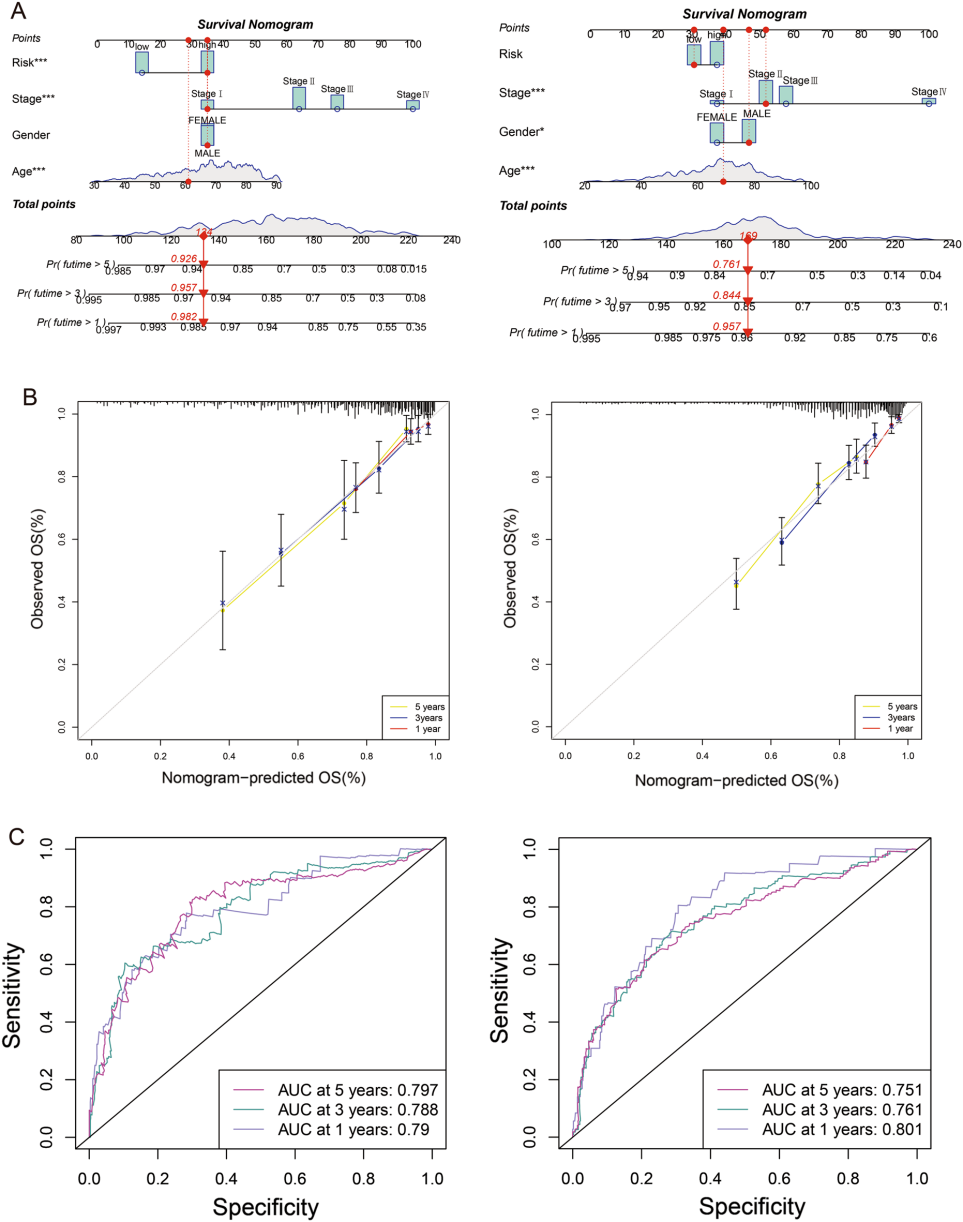
A nomogram was generated to estimate the survival rate of COAD patients. (**A**) Development of a nomogram by combining bile acid metabolism risk scores with age, gender, and TNM stage to predict the survival probability. (**B**) Calibration plots of the nomogram. (**C**) ROC curves of the nomogram. The left group represents TCGA-COAD cohort, and the right group represents GEO-COAD cohort.

A line graph was drawn depicting the factors involved in the construction of the nomogram, which included risk score, stage, age and gender. The line graphs could predict the 1-, 3- and 5-year OS of patients with COAD based on the total score (Fig. 3A). Furthermore, the calibration of the charts revealed that the predicted and actual values are in good agreement (Fig. 3B). Additionally, the transient ROC curve also reflects the high accuracy of the line graph in predicting survival (Fig. 3C).

### Immune-related characteristics and chemical response in the low- and high-risk score groups

**Figure 4:**
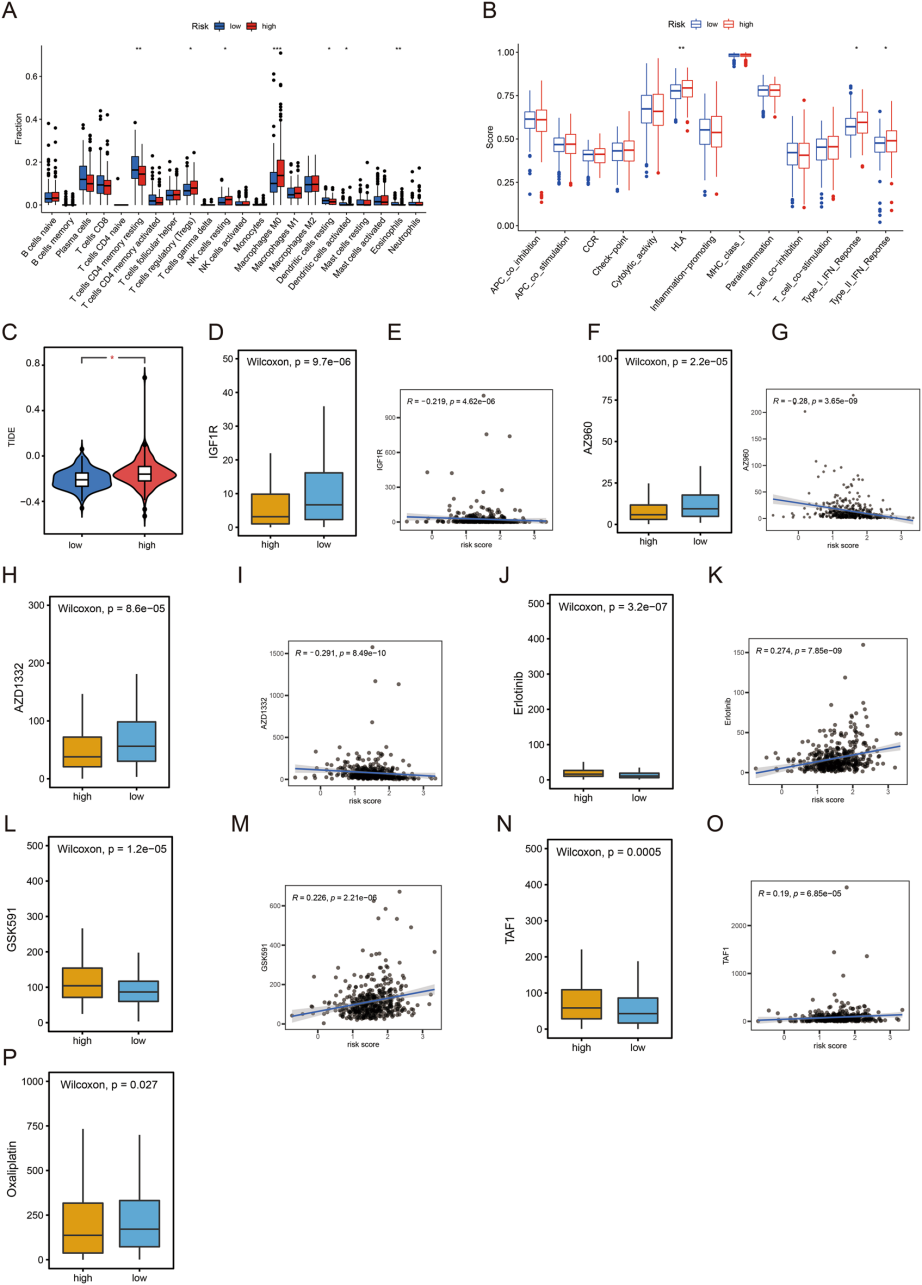
(**A**) The immune infiltration of immune cell types in high-risk and low-risk patients in the TCGA-COAD cohort. (**B**) Analysis of immune function in high-risk and low-risk patients in the TCGA-COAD cohort. ***p < 0.001, ns *p* > 0.05. (**C**) High-risk and low-risk COAD patients with TIDE scores in the TCGA-COAD cohort. (**D**–**P**) Bile acid metabolism score and IGF1R (**D**, **E**), AZ960 (**F**, **G**), AZD1332 (**H**, **I**), Erlotinib (**J**, **K**), GSK591 (**L**, **M**), TAF1 (**N**, **P**), Oxaliplatin (**O**) chemotherapeutic drug sensitivity analysis in the TCGA-COAD cohort.

To compare the immunological differences between the high and low-risk groups, we investigated both immune cells and immune function. The immune cells upregulated in the low-risk group were CD4 memory resting T cells, resting Dendritic cells, activated Dendritic cells and Eosinophils (*p* value < 0.05). Immune cells that were upregulated in the high-risk group were Tregs, resting NK cells and M0 Macrophages (*p* value < 0.05) (Fig. 4A). Immune function analysis (Fig. 4B) showed that the low-risk group was less active in HLA, Type I_IFN_Reponse and Type_II_IFN_Reponse than the high-risk group (*p *value < 0.05). TIDE analysis further revealed that the TIDE values of the high-risk group were higher than those of the low-risk group (Fig. 4C), indicating that the high-risk group had a higher chance of immune escape and that immunotherapy in these cases would be less effective. In the risk score and chemical sensitivity analysis, the drugs with significant differences between the high- and low-risk groups were screened (Supplementary Table S3). The top 3 differential drugs in the high-risk group were IGF1R, AZ960 and AZD1332 (Fig. 4D–I), while those in the low-risk group were Erlotinib, GSK591 and TAF1 (Fig. 4J–O). Oxaliplatin, a commonly used chemotherapeutic agent in a clinical setting for patients with COAD, was observed to be more sensitive in the high-risk group than the low-risk group (*p* value < 0.05) (Fig. 4P).

### PPI network of differentially expressed genes in the low- and high-risk groups

**Figure 5:**
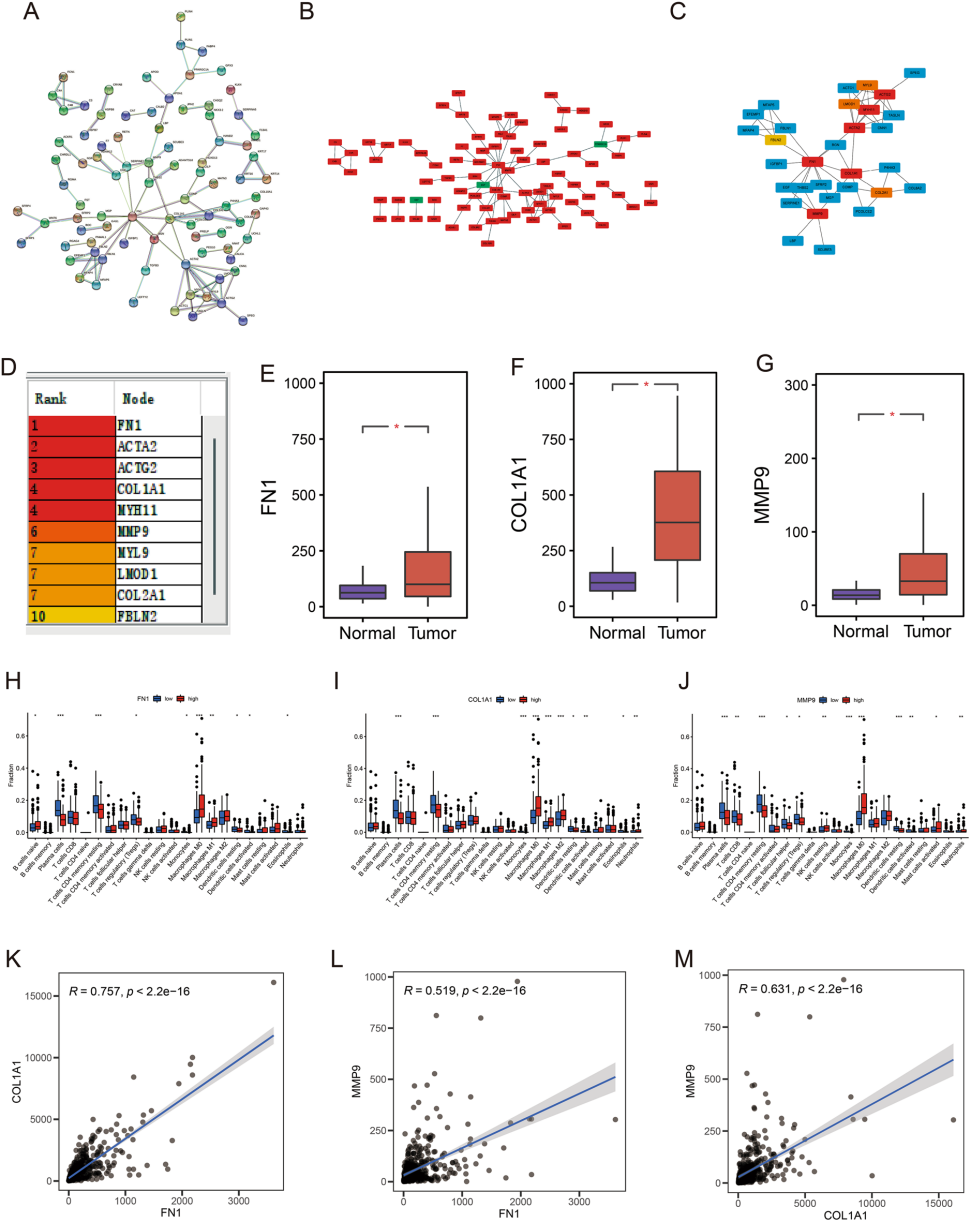
(**A**) PPI network of risk differential genes. (**B**) Distribution network diagram of up-regulated and down-regulated genes. (Red represents upregulated genes, green represents downregulated genes) (**C**, **D**) Top 10 hub genes of the gene expression network. (**E**–**G**) Comparison of FN1 (**E**), COL1A1 (**F**) and MMP9 (**G**) mRNA expression levels of each gene in COAD tissue and normal intestinal tissue. The expression levels of all genes in cancer tissues were higher than those in normal intestinal tissues. The red and blue boxes represent tumor and normal tissue, respectively. Red asterisks indicate significant differences in the expression of each mRNA (*p* value < 0.05). (**H**–**J**) Immune cell content of 22 immune cell types in FN1 (**H**), COL1A1 (**I**), and MMP9 (**J**) Hub genes. (**K**) FN1 and COL1A1 correlation analysis. (**L**) FN1 and MMP9 correlation analysis. (**M**) COL1A1 and MMP9 correlation analysis.

Using the STRING online database, a correlation analysis of the differential genes between the high- and low-risk groups was performed to construct a PPI network (Fig. 5A). The differential genes were visualized in Cytoscape with red representing upregulated genes and blue representing down-regulated genes (Fig. 5B). To identify hub genes, the cytoHubba plug-in was used, which revealed a total of 10 network core genes (*FN1, ACTA2, ACTG2, COL1A1, MYH11, MMP9, MYL9, LMOD1, COL2A1* and *FBLN2*) (Fig. 5C, D). Among these genes, three central genes (*FN1, COL1A1, and MMP9*) exhibited differential expression between the tumor and normal samples, as illustrated in Fig. 5E–G. These genes hold promise as candidate genes for further investigation in subsequent studies.

Immune cell infiltration analyses revealed that the upregulated immune cells in the *FN1* high expression group were naive B cells, M0 Macrophages and M1 Macrophages, whereas the downregulated cells were Plasma cells, CD4 memory resting T cells, Tregs, Monocytes, resting Dendritic cells, activated Dendritic cells and Eosinophils (Fig. 5H). The upregulated immune cells in the *COL1A1* high expression group were M0 Macrophages, M1 Macrophages, M2 Macrophages and Neutrophils, whereas the down-regulated immune cells were Plasma cells, CD4 memory resting T cells, Monocytes, resting Dendritic cells, activated Dendritic cells and Eosinophils (Fig. 5I). The immune cells that were upregulated in the *MMP9* high expression group were resting NK cells, M0 Macrophages, activated Mast cells and Neutrophils, whereas the downregulated immune cells were Plasma cells, CD8 T cells and CD4 memory resting T cells, Tregs, resting NK cells, Monocytes, resting Dendritic cells and activated Dendritic cells (Fig. 5J). Overall, the infiltration levels of the activated immune cells in the high-expression group were higher than that of the low-expression groups, indicating that these genes could be used as an indicator for suitability to immunotherapy. Furthermore, in the target gene correlation analysis, the three genes maintained a positive correlation with each other with statistically significant differences (*p* value < 0.001) (Fig. 5K–M).

## Discussion

COAD is one of the most common malignancies globally. Laparoscopic surgery combined with chemotherapy is often used to enhance the cure rate for patients with high-risk stage II and III disease^15^. Additionally, the current anatomically based traditional TNM staging is inefficient in predicting prognosis and has some limitations^16^. Contrarily, assessing patients with mid to late-stage COAD based on individual mutation statuses, such as KRAS and BRAF mutations, and MSI status offers more treatment opportunities^17^, However, the molecular level of COAD is very complex and relying on a single gene or factor to construct a predictive model would prove to be inefficient. Conversely, a robust and more accurate model can be constructed by combining multiple genes^18–20^. Therefore, we need a reliable prognostic gene signature to facilitate individualized prognosis and precision medicine treatment that can aid in predicting the survival of patients with COAD.

Tumour development is often influenced by the reprogramming of cellular metabolism. The extent of cancer activity can be demonstrated by changes in cellular metabolic activity^21^. For example, the upregulation of glycolytic metabolism suggests exacerbation of COAD^22,23^ while altered amino acid metabolism indicates metastasis, proliferation and drug resistance^24–26^. Normal bile acid metabolism contributes to the absorption of lipids, cholesterol and fat-soluble vitamins in the body and regulates the homeostasis of the epithelial environment of the gastrointestinal tract^27^. When bile acid is overproduced, it can cause abnormalities, such as oxidation, DNA damage, apoptosis and mutation^28^. Additionally, this high physiological level of bile acids can destabilize the genome at exposed sites, which can induce apoptosis and eventually lead to cancer^28^. Currently, to the best of our knowledge, there are no studies on COAD-related predictive models of bile acid metabolism. In this study, COAD data were mined from two databases: the TCGA-COAD cohort was used as the training set and the GEO-COAD cohort was used as the validation set. The construction of this new risk prediction model could aid in predicting the treatment outcome of individual patients, identifying target genes and analysing their infiltration in immune cells.

In this study, a strong correlation between genes related to bile acid metabolism and patients with COAD was observed, most notably in terms of OS. Furthermore, univariate and multifactorial Cox analyses revealed that age and risk scores could be used as independent predictors of OS. GO and KEGG enrichment analyses also showed that genes related to bile acid metabolism were closely associated with bile secretion, steroid and fatty acid metabolism and intestinal organic anion secretion. These genes also have the potential to act as signals and receptors that can influence cancer development and progression. The principle of immunotherapy is to inhibit cancer development by activating the natural immune molecular components of the tumour microenvironment^29^. Eosinophils indirectly generate type 1 T cell responses through the GM-CSF-IRF5 signalling axis, which has anti-tumour properties^30^. In the present study, the low-risk group was enriched in Eosinophils. This observation could be used in the treatment of patients with COAD, wherein Eosinophils could be used as an anti-tumour agent, especially in those with increased Eosinophil levels. According to previous studies, Tregs can produce cytokines to enhance anti-tumour properties^31^. The immune-related functions of Type_I_IFN_Reponse and Type_II_IFN_Reponse were active in the high-risk group. These responses are essential for the immune editing of tumours^32^, which could indicate that the high-risk group is more suitable for immunotherapy.

In the risk score and chemical sensitivity analysis, drugs with therapeutic differences in the high- and low-risk groups were obtained, which could provide new options for the clinical treatment of COAD. Oxaliplatin was more effective in the high-risk group, which is consistent with the current clinical use. Furthermore, ten core genes were identified between the high- and low-risk groups. The genes that showed increased expression in tumours were *FN1, COL1A1* and *MMP9* (*p* value < 0.05), which can be considered high-risk genes to indicate the prognosis level of patients with COAD. *FN1* is a highly enriched gene in human oesophageal cancer. Its expression is upregulated by SATB1 stimulation, causing oncogenic effects and inducing oesophagal cancer development^33^. Moreover, the HMGA2 transcriptional activation of *FN1* accelerates the progression of COAD^34^. Overexpression of COL1A1 in vivo can also lead to epithelial-mesenchymal transition, which leads to COAD metastasis^35^. *MMP-9* is a highly expressed gene in COAD, and its expression level is positively correlated with the pathological stage, lymph node metastasis and prognosis of patients^36^. The increased expression of *MMP9* is indicative of tumour progression through angiogenesis, invasion and metastasis, and it also causes the epithelial-mesenchymal transition, which shortens survival^37^. The results reported in the current study are consistent with previous reports, thus validating the accuracy of this study and the model’s scientific validity. Furthermore, the three central genes” ability to act on various immune cells suggests that immunotherapy could improve survival in patients with poor prognoses. However, although a positive correlation was observed between *FN1, COL1A1* and *MMP9* in COAD, further studies are required to validate this correlation.

## Conclusion

This study developed a novel risk model for bile acids metabolism based on data from the TCGA-COAD and GEO-COAD cohorts. A strong correlation between bile acid metabolism risk scores, immune cell infiltration, chemotherapy and immunotherapy effects was observed in patients with COAD. Furthermore, three target genes of bile acid metabolism (*FN1, COL1A1* and *MMP9*) were strongly associated with the clinical stage and prognosis of patients with COAD, with high specificity regarding immune cell infiltration. Therefore, to improve the prognosis of patients with COAD, *FN1, COL1A1* and *MMP9* can be used as biomarkers for the individualized treatment of patients with COAD.

### Supplementary Information

Supplementary Legends.

Supplementary Figure 1.

Supplementary Figure 2.

Supplementary Table S1.

Supplementary Table S2.

Supplementary Table S3.

## Data Availability

This study relied on publicly available data. The Cancer Genome Atlas (TCGA) and Gene Expression Omnibus (GEO) databases contain this information.

## References

[1] Siegel RL (2022). Cancer statistics, 2022. CA Cancer J. Clin..

[2] Zhuang Z (2021). Development and validation of a robust pyroptosis-related signature for predicting prognosis and immune status in patients with colon cancer. J. Oncol..

[3] Qiu Y (2022). Ferroptosis-related long noncoding RNAs as prognostic marker for colon adenocarcinoma. Appl. Bionics Biomech..

[4] Dumoulin FL, Hildenbrand R (2019). Endoscopic resection techniques for colorectal neoplasia: Current developments. World J. Gastroenterol..

[5] Benson AB (2020). NCCN guidelines insights: rectal cancer, version 6.2020. J. Natl. Compr. Canc. Netw..

[6] Chiang JY (2013). Bile acid metabolism and signaling. Compr. Physiol..

[7] Fiorucci S (2020). Bile acid modulators for the treatment of nonalcoholic steatohepatitis (NASH). Expert Opin. Investig. Drugs.

[8] Rajani C, Jia W (2018). Bile acids and their effects on diabetes. Front. Med..

[9] Fiorucci S (2021). Bile acid signaling in inflammatory bowel diseases. Dig. Dis. Sci..

[10] Jia W, Xie G, Jia W (2018). Bile acid-microbiota crosstalk in gastrointestinal inflammation and carcinogenesis. Nat. Rev. Gastroenterol. Hepatol..

[11] Fu T (2019). FXR regulates intestinal cancer stem cell proliferation. Cell.

[12] Wang S (2019). Interplay between bile acids and the gut microbiota promotes intestinal carcinogenesis. Mol. Carcinog..

[13] Farhana L (2016). Bile acid: A potential inducer of colon cancer stem cells. Stem Cell Res. Ther..

[14] Tabe Y, Konopleva M, Andreeff M (2020). Fatty acid metabolism, bone marrow adipocytes, and AML. Front. Oncol..

[15] Miller KD (2016). Cancer treatment and survivorship statistics, 2016. CA Cancer J. Clin..

[16] Sjöblom T (2006). The consensus coding sequences of human breast and colorectal cancers. Science.

[17] Hegde M (2014). ACMG technical standards and guidelines for genetic testing for inherited colorectal cancer (Lynch syndrome, familial adenomatous polyposis, and MYH-associated polyposis). Genet. Med..

[18] Zhang Q (2020). Weighted correlation gene network analysis reveals a new stemness index-related survival model for prognostic prediction in hepatocellular carcinoma. Aging (Albany NY).

[19] Xue Y, Li J, Lu X (2020). A novel immune-related prognostic signature for thyroid carcinoma. Technol. Cancer Res. Treat..

[20] Bao M, Zhang L, Hu Y (2020). Novel gene signatures for prognosis prediction in ovarian cancer. J Cell Mol Med.

[21] Pavlova NN, Thompson CB (2016). The emerging hallmarks of cancer metabolism. Cell Metab..

[22] DeBerardinis RJ, Chandel NS (2016). Fundamentals of cancer metabolism. Sci. Adv..

[23] Wei X (2019). DT-13 inhibited the proliferation of colorectal cancer via glycolytic metabolism and AMPK/mTOR signaling pathway. Phytomedicine.

[24] Prosén S (2022). Increased expression of LAT1 in basal cell carcinoma: implications for tumour cell survival. Clin. Exp. Dermatol..

[25] Hua Q (2021). CEMIP, a novel adaptor protein of OGT, promotes colorectal cancer metastasis through glutamine metabolic reprogramming via reciprocal regulation of β-catenin. Oncogene.

[26] Pranzini E (2021). Metabolic reprogramming in anticancer drug resistance: A focus on amino acids. Trends Cancer.

[27] Fiorucci S (2010). Counter-regulatory role of bile acid activated receptors in immunity and inflammation. Curr. Mol. Med..

[28] Payne CM (2008). Hydrophobic bile acids, genomic instability, Darwinian selection, and colon carcinogenesis. Clin. Exp. Gastroenterol..

[29] Huang D (2022). Characteristics of fatty acid metabolism in lung adenocarcinoma to guide clinical treatment. Front. Immunol..

[30] Arnold IC (2020). The GM-CSF-IRF5 signaling axis in eosinophils promotes antitumor immunity through activation of type 1 T cell responses. J. Exp. Med..

[31] Ferreira C (2020). Type 1 T(reg) cells promote the generation of CD8(+) tissue-resident memory T cells. Nat. Immunol..

[32] Di Franco S (2017). Role of type I and II interferons in colorectal cancer and melanoma. Front. Immunol..

[33] Song G (2017). SATB1 plays an oncogenic role in esophageal cancer by up-regulation of FN1 and PDGFRB. Oncotarget.

[34] Wu J (2016). Transcriptional activation of FN1 and IL11 by HMGA2 promotes the malignant behavior of colorectal cancer. Carcinogenesis.

[35] Zhu X (2020). Bone morphogenetic protein 1 targeting COL1A1 and COL1A2 to regulate the epithelial-mesenchymal transition process of colon cancer SW620 cells. J. Nanosci. Nanotechnol..

[36] Ruan P (2020). Expression and clinical significance of CD74 and MMP-9 in colon adenocarcinomas. J. BUON.

[37] Buttacavoli M (2021). Integrated multi-omics investigations of metalloproteinases in colon cancer: Focus on MMP2 and MMP9. Int. J. Mol. Sci..

