# Supplementary material for: Construction and validation of a prognostic model for colon adenocarcinoma based on bile acid metabolism-related genes
Source: Sci Rep. 2023 Aug 5;13:12728. doi: 10.1038/s41598-023-40020-z (PMC10404223; doi:10.1038/s41598-023-40020-z)
Supplement: Supplementary file 1 — Supplementary Legends. [file 41598_2023_40020_MOESM1_ESM.docx]

Supplementary figure 1. GO and KEGG enrichment analysis of the up-regulated DEGs and down-regulated DEGs. (A) KEGG pathway analysis of up-regulated DEGs. (B) KEGG pathway analysis of down-regulated DEGs. (C) GO pathway analysis of up-regulated DEGs. (D) GO pathway analysis of down-regulated DEGs. BP represents the biological process, CC represents the cellular component, and MF represents the molecular function.

Supplementary figure 2. The distribution of risk scores, survival status of patients and the heatmap of gene expression profiles in GEO-COAD-dataset2. (A) The distribution of survival status of COAD patients with increasing bile acid metabolism risk scores. (B) The distribution of bile acid metabolism risk scores of patients. (C) Heatmap for the expression of six crucial genes in GEO-COAD-dataset2. (D) Kaplan-Meier curves for low- and high-risk groups in GEO-COAD-dataset2.

Supplementary table S1. Genes related to bile acid metabolism.

Supplementary table S2. Drugs with significant differences between the high- and low-risk groups.
