# Supplementary figures and images for: Construction and validation of a prognostic model for colon adenocarcinoma based on bile acid metabolism-related genes
Source: Sci Rep. 2023 Aug 5;13:12728. doi: 10.1038/s41598-023-40020-z (PMC10404223; doi:10.1038/s41598-023-40020-z)

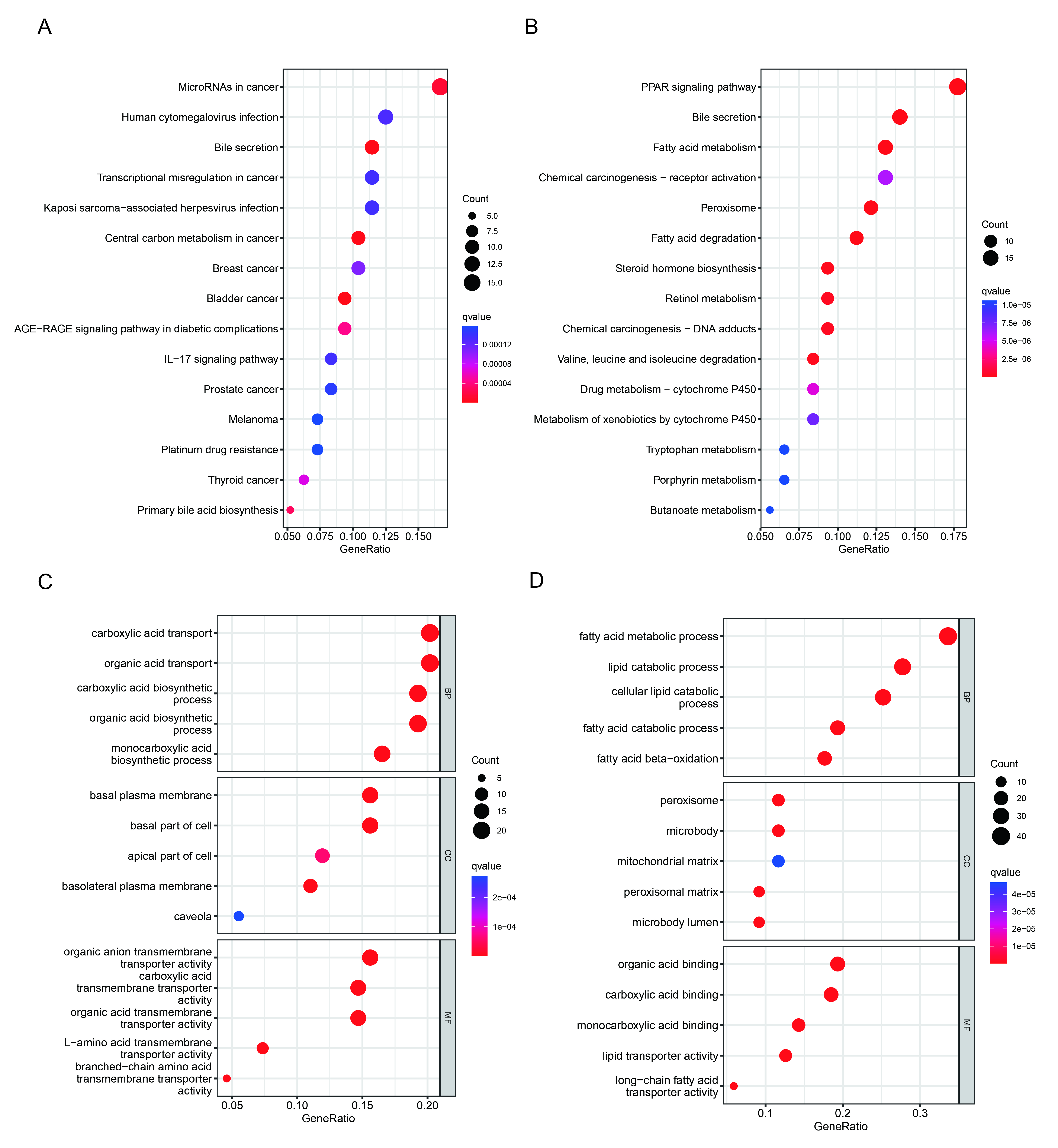

Supplement: Supplementary file 2 — Supplementary Figure 1. [file 41598_2023_40020_MOESM2_ESM.jpg]

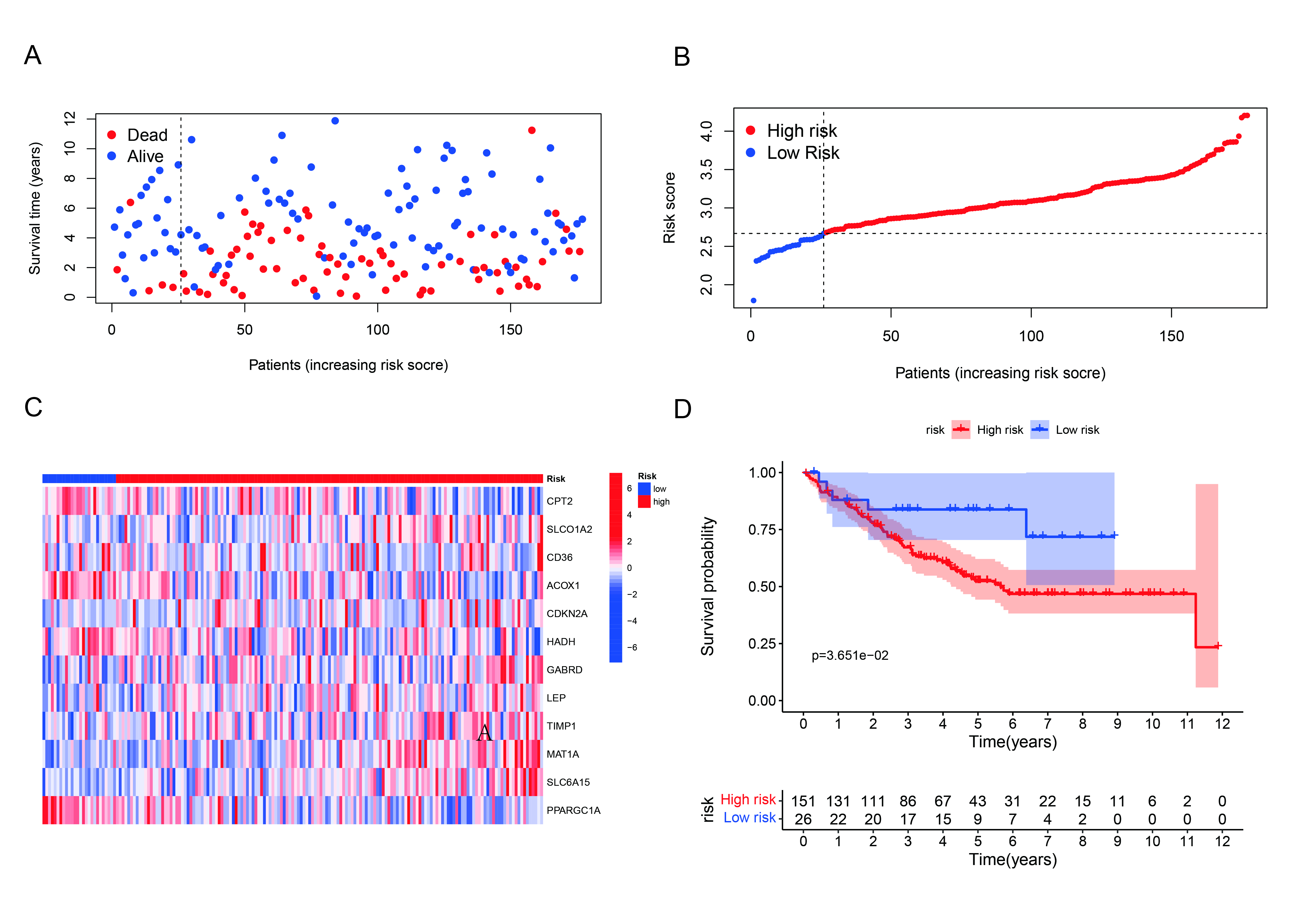

Supplement: Supplementary file 3 — Supplementary Figure 2. [file 41598_2023_40020_MOESM3_ESM.jpg]
